# Tackling heterogeneity in treatment-resistant breast cancer using a broad-spectrum therapeutic approach

**DOI:** 10.20517/cdr.2022.40

**Published:** 2022-10-12

**Authors:** Leroy Lowe, J. William LaValley, Dean W. Felsher

**Affiliations:** ^1^Getting to Know Cancer (NGO), Truro, Nova Scotia B2N 1X5, Canada.; ^2^Wellness Plan MD Ltd, Austin, TX 78759, USA.; ^3^Division of Oncology, Departments of Medicine and Pathology, Stanford University, CA CCSR 1105, USA.

**Keywords:** Breast cancer, chemoresistance, drug resistance, targeted therapy

## Abstract

Tumor heterogeneity can contribute to the development of therapeutic resistance in cancer, including advanced breast cancers. The object of the Halifax project was to identify new treatments that would address mechanisms of therapeutic resistance through tumor heterogeneity by uncovering combinations of therapeutics that could target the hallmarks of cancer rather than focusing on individual gene products. A taskforce of 180 cancer researchers, used molecular profiling to highlight key targets responsible for each of the hallmarks of cancer and then find existing therapeutic agents that could be used to reach those targets with limited toxicity. In many cases, natural health products and re-purposed pharmaceuticals were identified as potential agents. Hence, by combining the molecular profiling of tumors with therapeutics that target the hallmark features of cancer, the heterogeneity of advanced-stage breast cancers can be addressed.

Breast cancer is a consequence of complex epigenetic and genetic alterations. The heterogeneity and evolution within breast cancers underpin tumor progression, as well as therapeutic resistance^[1-3]^. In recent years, significant efforts have focused on addressing therapeutic options that can address tumor heterogeneity in breast cancer^[4-6]^. This is particularly important in triple-negative breast cancers and metastatic breast cancers because many patients with advanced breast cancers will succumb to their disease as tumor heterogeneity gives rise to therapeutic resistance^[7-9] ^[Figure 1].

**Figure 1:**
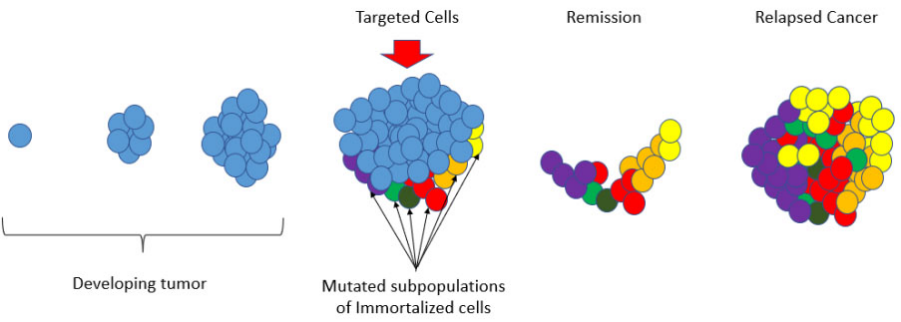
Therapeutic resistance - Developing tumors begin with a single immortalized cell that may have a single therapeutic target that can act to stop those cells from replication. For example, Tamoxifen (TAM) is the most common therapy used for the treatment of estrogen receptor-positive (ER+) breast cancer and it is used successfully in many cases. However, there are advanced-stage breast cancers that are plagued with mutated subpopulations of immortalized cells and cancer stem cells that play a key role in breast cancer progression, and metastasis. In these cancers, a single targeted therapy may produce a remission by successfully arresting some of the immortalized cells that have been targeted. However, if the remaining subpopulations of cells are driven by different mechanisms and prove to be chemoresistant, they will persist during remission and ultimately produce a relapsed cancer that is fully refractory to the initial treatment or combination of treatments.

To address the challenge of therapeutic resistance and tumor heterogeneity, a group of 180 cancer researchers collaborated in “The Halifax Project” to consider combinations of agents that might be employed^[10]^. In this effort, twelve teams of researchers were organized around the Hallmarks of Cancer^[11]^ and tasked to identify high-priority targets along with corresponding therapeutic agents that could reach those targets with limited toxicity. The hallmarks used are attributes ultimately found in most cancers (i.e., genomic instability, sustained proliferative signaling, tumor-promoting inflammation, evasion of anti-growth signaling, resistance to apoptosis, replicative immortality, dysregulated metabolism, immune system evasion, angiogenesis, tissue invasion and metastasis, and an accommodating tumor micro-environment). The overarching goal was to identify a significant number of agents that have limited to no toxicity, that might be combined to reach a multitude of key targets simultaneously.

Cancer is caused by an array of mutations and genomic events that coordinate to activate aberrant pathways. To address this complexity, we used the Hallmarks of Cancer as an organizing framework. We then focused on the development of a "broad-spectrum" methodology and therapeutic agents that could be combined using this approach. In particular, we focused on identifying natural health products (NHPs) and re-purposed pharmaceuticals, because both are readily available, often well tolerated, and broadly applicable to many cancers^[10,12-22]^. A detailed rationale for the methodology was provided and it was determined that it should be feasible from a safety standpoint and relatively inexpensive to implement^[23]^.

In the table below [Table 1] we have provided a sampling of NHP with updated references to show how these agents act on key mechanisms and pathways across the hallmarks of cancer^[10-22]^.

**Table 1:** Aligning targets with the hallmarks of cancer

**Cancer hallmark**	**Examples of potential agents**	**Key mechanism or pathway**
Genomic instability	Allyl Isothiocyanate^[24]^ Chrysin^[25]^ Plumbagin^[26]^	DNA damage and condensation DNA double-strand break repair DNA damage
Sustained proliferative signaling	Resveratrol^[27]^ Perillyl alcohol^[28]^ Artemisinin^[29]^	Cell cycle Cell cycle Cell cycle
Tumor-promoting Inflammation	Rosmarinic acid^[30]^ Berberine^[31]^ Curcumin^[32]^ Punica granatum L^[33]^	NF3 kappaB-p53-caspase-3 pathways NLRP3 Inflammasome pathway Nuclear factor-κB (NF-κB) miRNA-27a and miRNA-155
Evasion of anti-growth signaling	Deguelin^[34]^ Luteolin^[35]^ Withaferin A^[36,37]^ Curcumin^[38]^	EGFR-p-AKT/c-Met p-ERK AKT/mTOR pathway Notch2 SLC1A5-mediated ferroptosis
Resistance to apoptosis	EGCG^[39]^ Gossypol^[40]^ Triptolide^[41]^ Kaempferol^[42]^ Berberine^[43,44]^	P53/Bcl-2 pathway miRNA expression of many apoptosis‑related genes p38/Erk/mTOR Bcl2 Bcl2 (and many other pathways)
Replicative immortality	Curcumin^[45,46]^ Silibinin^[46]^ Coumestrol^[47]^ Diosmin^[48]^	Telomerase expression Telomerase expression Protein kinase CKII Senescence
Dysregulated metabolism	Resveratrol^[49]^ Metformin^[50]^ Baicalein^[51]^ Carpesium abrotanoides L.^[52]^	6-phosphofructo-1-kinase HIF-1alpha HIF-1alpha Glucose Metabolism and PKM2/HIF-1alpha axis
Immune system evasion	Astragalus polysaccharides^[53]^ Cordycepin^[54]^ Resveratrol^[55]^	Macrophage activation IL-2, TGF-β, IL-4 MICA/B and natural killer cells
Angiogenesis	Curcumin^[56]^ EGCG^[57]^ Melatonin^[58,59]^ Resveratrol^[60]^	NF-κB pathway VEGF VEGF VEGF
Tissue invasion and metastasis	Diallyl trisulfides^[61]^ Resveratrol^[62]^ Anthocyanins^[63]^ Cordycepin^[64]^	HIF-1alpha TGF-beta1 / Epithelial-Mesenchymal Transition FAK Hedgehog pathway
Tumor micro-environment	Resveratrol^[65]^ Sulforaphane^[66,67]^	Macrophage polarization Adipose mesenchymal stem cells

FAK: Focal adhesion kinase; EGCG: epigallocatechin gallate; VEGF: vascular endothelial growth factor.

Some NHPs, such as curcumin and resveratrol, target multiple signaling networks and pathways simultaneously which is attractive molecular promiscuity^[68,69]^. The appropriate selection of NHPs can also offer synergies for chemotherapy and radiation therapy treatment. For example, curcumin, resveratrol, tocotrienol, garcinol and quercetin have a mechanism of action that increases chemosensitivity^[70,71]^ and can reduce chemoresistance. While other NHPs, such as ellagic acid, diindolylmethane, and berberine, can increase radiation sensitivity^[72-74]^.

Other NHPs, such as gingerol and curcumin can also protect normal (healthy) cells from adverse toxicity from cytotoxic agents^[75,76]^. Thus, NHPs have many features that may designate them particularly suited as agents that might be used in situations where tumor heterogeneity has resulted in chemoresistance.

In particular, for breast cancer, the presence of subpopulations of cancer stem cells (CSCs) is known to be one cause of chemo-resistance and ultimately contributes to therapeutic relapse^[77]^. Notably, there are specific NHPs such as sulforaphane, curcumin, genistein, resveratrol, lycopene, and epigallocatechin-3-gallate that have been shown to promote cell cycle arrest and apoptosis in triple-negative breast cancer cells and which have also been shown to inhibit important CSC pathways, such as NF-κB, PI3K/Akt/mTOR, Notch 1, Wnt/β-catenin, and YAP^[78]^.

In addition to NHPs, there are also many existing pharmaceuticals that could provide additional targeting options. Numerous commonly prescribed non-oncology drugs possess multi-targeted anti-cancer effects. Pharmaceuticals already on the market have significant safety records and robust drug-drug interaction data compared to natural products and several researchers have looked at the effects of existing pharmaceuticals as it relates to relapse. Retsky (2012, 2020), for example, observed that the perioperative use of the NSAID analgesic ketorolac appears to reduce early relapse following mastectomy in breast cancer^[79,80]^. Hence, the use of both NHPs and repurposed pharmaceuticals to reach a broad-spectrum of molecular targets could be useful in developing personalized treatment protocols^[81] ^[Figure 2].

**Figure 2:**
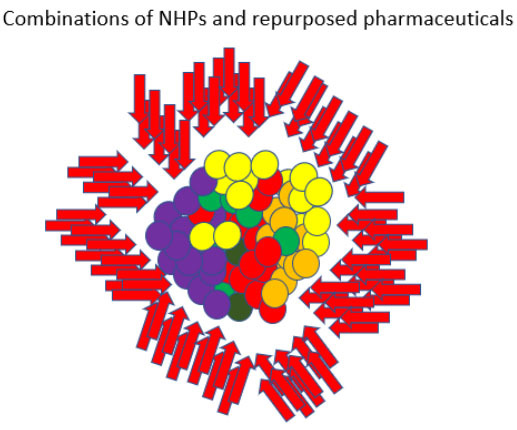
A broad-spectrum approach - In a broad-spectrum approach, heterogeneous subpopulations of chemoresistant immortalized cells are not targeted using a single targeted therapy or even a combination of 2-3 chemotherapy agents. Instead, a significant number of low toxicity agents are aimed at a multitude of key pathways/mechanisms simultaneously. Since most immortalized cells are driven by the pathways/mechanisms described in the Hallmarks of Cancer framework^[11]^, this approach increases the chance that a significant number of synergistic effects will be produced (i.e., since each affected cell will potentially be acted on in a multitude of ways).

Although our proposed approach has many potential advantages, there are challenges to conducting validating clinical studies. First, there is a shortage of funding for this type of initiative due to lack of patentability, manufacturing difficulties, contamination, and lack of product consistency^[82]^. Second, the use of NHPs among cancer patients is quite common. In fact, many patients who use them do not share the details with their physicians because they feel their physicians are not knowledgeable or will be indifferent or negative toward their use^[83,84]^. Finally, NHPs have yet to be approved by the FDA and although many NHPs are available as supplements and generally well-tolerated over an extended duration, the clinical evidence for these agents is often weak or non-existent.

One example of how NHPs can be used to improve the treatment of breast cancer is that NHPs have been shown to combine with Tamoxifen synergistically in inhibition of tumor cell growth, improved Tamoxifen sensitivity and reduction of Tamoxifen side effects^[85]^. However, some NHPs showed estrogen-like activity, which could reduce the effect of Tamoxifen, underscoring the need for a detailed analysis of any protocol that combines a multitude of agents^[85]^. They did find that some NHPs (e.g., morin, silybin, epigallocatechin gallate, myricetin, baicalein, curcumin, kaempferol, and quercetin) helped to increase the bioavailability of Tamoxifen *in vivo*. These promising observations suggest that NHPs with Tamoxifen are worthy of clinical studies.

Some NHPs are used to support cancer therapy by clinicians who practice integrative oncology; however, these physicians are typically less familiar with the molecular mechanisms of cancer signaling^[86]^. Instead, integrative oncology mainly focuses on the treatment of cancer-related symptoms such as acupuncture for nausea, exercise for sleep, and anxiety^[86]^. Indeed, a survey of clinics in Washington State showed that more than 72 oral or topical, nutritional, botanical, fungal and bacterial-based medicines had been used during the first year of care of the female breast cancer patients studied (*n *= 324)^[87]^. Since most of these agents are not aimed at the molecular mechanisms of cancer, the use of NHPs for these purposes would typically not include the type of analysis that would be needed to target tumor heterogeneity.

We highlight this approach as an important avenue that should be investigated further because the idea of reaching many key targets simultaneously makes sense given what is now known about the biology of cancer. Importantly, this is not something that has been attempted previously. Clinical trials of NHPs that have been undertaken typically involve single agents or limited combinations of agents at best. We speculate the use of combinations of NHPs that are able to hit multiple targets is most likely to be clinically effective.

The goal should be to provide a clinical treatment protocol that makes a rational utilization of the evidence base. If this approach is to work, future efforts utilizing NHPs and repurposed pharmaceuticals will require clinical studies involving unique combinations of dozens of agents in protocols that are tailored/personalized for each patient. The agents that are used will therefore need to be carefully considered for potential interactions, and some of these agents may have shown limited or no activity when used individually. Until there is clinical research that fully explores the synergies that can be produced when a significant number of pathways are targeted simultaneously, the true potential of combining these actions all at once will simply not be known.

Finally, we acknowledge that what we are proposing would require a change from phased clinical trials. A case series or a cohort study might be a more appropriate means to document the results of experimental efforts of this nature^[88]^, since each patient will require an individualized protocol. We do believe that such an approach may be able to help address the challenges of therapeutic resistance that emerge in many breast cancers.

## DECLARATIONS

### Copyright

© The Author(s) 2022.
